# Complete genome sequence of the polyethylene terephthalate-degrading bacterium *Pseudideonella sakaiensis* strain 201-F6, formerly known as *Ideonella sakaiensis*

**DOI:** 10.1128/mra.00682-26

**Published:** 2026-09-21

**Authors:** Jonas Koh, Bin Cao

**Affiliations:** 1 Singapore Centre for Environmental Life Sciences Engineering, Nanyang Technological University 54761, Singapore, Singapore; 2 Centre for Animal and Veterinary Sciences, National Parks Board (NParks) 68732, Singapore, Singapore; 3 The Gene and Linda Voiland School of Chemical Engineering and Bioengineering, Washington State University 6760, Pullman, Washington, USA; Rochester Institute of Technology, Rochester, New York, USA

**Keywords:** *Piscinibacter sakaiensis*, *Ideonella sakaiensis*, polyethylene terephthalate, PET degradation, complete genome, plasmid, *Pseudideonella sakaiensis*

## Abstract

Using hybrid Illumina and Oxford Nanopore sequencing, we report the first complete, circularized genome of *Pseudideonella sakaiensis* strain 201-F6, originally described as *Ideonella sakaiensis* and subsequently reclassified as *Piscinibacter sakaiensis*: 6,051,261-bp chromosome and 128,612-bp plasmid. It supports molecular studies and highlights an undercharacterized plasmid whose coding DNA sequence are largely poorly annotated.

## ANNOUNCEMENT

*Pseudideonella sakaiensis* (1), originally described as *Ideonella sakaiensis* and subsequently reclassified as *Piscinibacter sakaiensis*, is a gram-negative bacterium that attracted attention for its ability to utilize polyethylene terephthalate (PET) and PET-derived compounds as carbon and energy sources (2). Strain 201-F6 was originally isolated from PET-contaminated sediment and has become a model organism for studying plastic degradation and enzymatic depolymerization mechanisms. Despite its importance, previously published genomes (GCF_001293525.1 [2] and GCF_055361595.1 [3]) remain at the contig level, lacking chromosome circularization, which limits resolution of operons, promoters, and synteny. Here, we present a complete, circularized genome of *P. sakaiensis* 201-F6.

Strain 201-F6 (NBRC 110686) was obtained lyophilized from the Bioresource Collection and Research Center and reconstituted in NBRC 802 medium (10 g/L hipolypepton, 2 g/L yeast extract, 1 g/L MgSO_4_·7H_2_O) at 30°C. Genomic DNA was extracted using the PureLink Genomic DNA Mini Kit. Whole-genome sequencing was performed using the Illumina and Oxford Nanopore Technologies (ONT) platforms. Illumina libraries were prepared with the TruSeq DNA Nano Library Preparation Kit and dual-barcoded IDT TruSeq DNA UD Indexes and then sequenced on a HiSeqX v2.5 platform (2 × 150 bp). ONT libraries were prepared with SQK-NBD114 Native Barcoding Kit (V14) and sequenced on a GridION using an R10.4.1 MinION flow cell.

Illumina reads were trimmed using Trimmomatic (v0.39-2) (4) and quality-checked with FastQC (v0.12.1) (5), retaining 71,294,055 (9.8 Gb) paired-end reads. ONT reads were base-called with Dorado (high-accuracy model, v7.3.11). Because pre-assembly quality checks detected residual adapter sequences in a subset of Dorado base-called reads, Porechop (v0.2.4) (6) was used only for residual adapter removal. Reads were then filtered for length (>10 kb) and quality (top 90%) using FiltLong (v0.2.1) (7), yielding 77,421 reads (1.22 Gb, N50 of 15.9 kb, mean quality 13.1). Long reads were assembled with Flye (v2.9.4) (8) and polished with Medaka (v1.12.0). The assembly was provided to Unicycler (v0.5.1) (9) via “--existing_long_read_assembly” for graph resolution and circularization, followed by short-read polishing with polypolish (v0.6.0) (10) and pypolca (v0.3.1) (11, 12).

The final assembly comprised one circular chromosome (6,051,261 bp; 73.0% GC, based on National Center for Biotechnology Information [NCBI] Prokaryotic Genome Annotation Pipeline [PGAP] annotation) and one circular plasmid (128,612 bp; 66.5% GC), with ONT and Illumina coverages of 196× and 1,586×, respectively. Although a recent study recovered a ~ 128 kbp plasmid (3), its chromosome remained fragmented at the contig level (GenBank accession GCA_055361595.1). Here, we provide a complete circularized assembly resolving both the chromosome and the plasmid. Genome quality assessment using CheckM2 (v1.1.0) (13) on Galaxy with the Diamond database (v1.0.2) (14) showed 100% completeness and 1.29% contamination. GTDB-Tk (v1.7.0) (15) confirmed the isolate as *P. sakaiensis* (FastANI = 99.97% vs draft genome GCF_001293525.1). All 227 contigs of the draft genome mapped to our assembly, with 64 mapping more than once. Annotation using PGAP v6.8 (16) identified 5,570 coding sequences, 6 complete ribosomal RNA (rRNA) genes (2 copies each of 5S, 16S, and 23S rRNA), 48 transfer RNA genes, and 3 non-coding RNAs. This complete, plasmid-resolved genome provides a high-resolution reference for studying PET degradation pathways, gene regulation, and extrachromosomal elements in *P. sakaiensis*.

## Data Availability

The complete and circularized genome has been submitted to NCBI with the RefSeq assembly GCF_056715965.1 (BioSample SAMN41414263), under the BioProject PRJNA1112095. Unassembled raw Illumina and Oxford Nanopore Technologies reads are found under the Sequence Read Archive numbers SRR39038256 and SRR39038255, respectively.
